# Advancements in Genomic and Behavioral Neuroscience Analysis for the Study of Normal and Pathological Brain Function

**DOI:** 10.3389/fnmol.2022.905328

**Published:** 2022-06-23

**Authors:** Annalisa M. Baratta, Adam J. Brandner, Sonja L. Plasil, Rachel C. Rice, Sean P. Farris

**Affiliations:** ^1^Center for Neuroscience, School of Medicine, University of Pittsburgh, Pittsburgh, PA, United States; ^2^Department of Pharmacology & Chemical Biology, School of Medicine, University of Pittsburgh, Pittsburgh, PA, United States; ^3^Department of Anesthesiology and Perioperative Medicine, School of Medicine, University of Pittsburgh, Pittsburgh, PA, United States; ^4^Department of Biomedical Informatics, School of Medicine, University of Pittsburgh, Pittsburgh, PA, United States

**Keywords:** genomics, genetics, behavior, neuroscience, technology

## Abstract

Psychiatric and neurological disorders are influenced by an undetermined number of genes and molecular pathways that may differ among afflicted individuals. Functionally testing and characterizing biological systems is essential to discovering the interrelationship among candidate genes and understanding the neurobiology of behavior. Recent advancements in genetic, genomic, and behavioral approaches are revolutionizing modern neuroscience. Although these tools are often used separately for independent experiments, combining these areas of research will provide a viable avenue for multidimensional studies on the brain. Herein we will briefly review some of the available tools that have been developed for characterizing novel cellular and animal models of human disease. A major challenge will be openly sharing resources and datasets to effectively integrate seemingly disparate types of information and how these systems impact human disorders. However, as these emerging technologies continue to be developed and adopted by the scientific community, they will bring about unprecedented opportunities in our understanding of molecular neuroscience and behavior.

## Introduction

The study of psychiatric and neurological disorders has presented a problem for researchers trying to understand the etiology of these conditions. While many of the diseases are uniquely human, the majority of molecular studies required to understand the biological underpinnings of these pathologies cannot readily be performed in a clinical setting. In order to make advancements in understanding disease, most laboratories turn to model systems and organisms, which allow for greater degree of experimental control and in-depth analysis. Recent developments, such as the discovery of clustered regularly interspaced short palindromic repeats (CRISPR), have increased the ease with which individual laboratories can create model systems. To keep up with advances in gene editing technology, new techniques for studying the genome and behavior of novel animal models are continually being developed.

CRISPR/Cas9 was originally discovered in the bacterium *Streptococcus pyogenes* (Bhaya et al., 2011). It was quickly recognized that this system was much less time consuming and more cost-effective than previous gene editing techniques such as zinc finger nucleases (ZFNs) and transcription activator-like effector nucleases (TALENs) (Gupta and Musunuru, 2014). The timeline for the creation of genetically engineered animals is also much shorter, with the average timeline to create knockout animals being less than a year (Homanics, 2019), although accelerated methods have produced full experimental cohorts in a single generation (Plasil et al., 2020). Beyond its ability to create double stranded breaks in DNA and inactivate genes, CRISPR genome editing has a number of additional uses, including the creation of genetic knock-in animals (Yang et al., 2013; Aida et al., 2015; Renaud et al., 2016), activating or repressing transcription (Liao et al., 2017; Zheng et al., 2018), and direct targeting of RNA rather than DNA (Cox et al., 2017). The versatility of CRISPR also allows for it to be used in a number of model systems and organisms both in *in vitro* and *in vivo*, from genetically engineering cells in a dish to rodents, non-human primates, and other species (Kang et al., 2019; Plasil et al., 2020). The ease with which the CRISPR system can be used in a laboratory has opened the door for research groups to quickly and easily create model systems and organisms for specialized study.

With the development of new model systems and organisms, researchers have also developed a number of new techniques for characterizing molecular and behavioral adaptations. As part of this review, we will discuss advancements in high-throughput genomic approaches and behavioral neuroscience that allow for closer and more accurate characterization of model systems. Advancements in genomic technologies have allowed researchers to move beyond observing changes in overall gene expression on a tissue level and begin to investigate cell-type and region-specific changes. Additionally, the integration of machine learning into the assessment of behavioral phenotypes allows for the observation of very fine behavioral changes, such as eye movements or gait, while also reducing the amount of time and possible bias that is introduced by experimenter observations. While powerful tools when used separately, the integration of these two techniques will allow for incredibly in-depth analysis of model systems and organisms, greatly advancing our understanding of how the healthy brain functions, as well as the etiology and treatment of psychiatric and neurological diseases.

## Advances in Genomic Technology for Transcriptomic, Translatomic, and Epigenomic Analysis

### Current High-Throughput Sequencing Technology

Sequencing technologies were developed in the late 1970s and revolutionized the study of genetics. Sanger Sequencing was the first application to automate DNA-sequencing using chain-terminating and radioactively labeled or fluorescently labeled dideoxynucleotides to sequence complementary DNA to a template strand (Sanger et al., 1977). This method, categorized as ‘first-generation sequencing,’ was continuously modified and improved to sequence genomes (Hu et al., 2021). Sanger Sequencing aided in the human genome project, which took over a decade to complete and an initial investment of $2.7 billion (National Human Genome Research Institute, 2021). However, this technique had limited throughput for whole-genome analyses due to sequential sequencing performing one reaction at a time.

Since the human genome was initially sequenced there has been substantial improvements that have significantly reduced the time and cost of sequencing (Marioni et al., 2008; Wheeler et al., 2008; Li et al., 2014; Seqc/Maqc-Iii Consortium, 2014). Following ‘first-generation sequencing’ the next wave of sequencing technologies have been referred to as ‘next-generation sequencing’ (NGS) (Wheeler et al., 2008). NGS increased throughput by allowing for parallel sequencing of nucleotides (Li et al., 2014; Seqc/Maqc-Iii Consortium, 2014; Chatterjee et al., 2018). NGS is capable of generating a high volume of data, increased efficiencies, and a wide-range of technical and biological applications. NGS can be divided into two complementary nucleotide based-techniques, DNA sequencing and RNA sequencing (DNA-Seq and RNA-Seq). As these names suggest DNA-Seq techniques are specific to the genome, whereas RNA-Seq techniques are used to study the transcriptome [for more detailed reviews: (Stark et al., 2019; Hong et al., 2020; Hu et al., 2021)].

The general NGS protocol used for both DNA-Seq and RNA-Seq includes DNA fragmentation, end-repair, adaptor-ligation, and PCR amplification, to generate a library of diverse cDNA fragments (Mortazavi et al., 2008; Chatterjee et al., 2018), although cDNA synthesis is not required for DNA-Seq. DNA-Seq encompasses a variety of sequencing options dependent on the template: Whole Genome Sequencing (WGS), Whole Exome Sequencing (WES), Epigenome Sequencing, and Targeted Sequencing (Rizzo and Buck, 2012). WGS sequences the entire genome, whereas WES is restricted to exonic coding regions. Epigenome Sequencing studies changes in the epigenome, enriching for changes in chromatin assembly, chromatin interaction, and histone modifications (e.g., methylation and acetylation). Targeted Sequencing is restricted to a specific set of genes or genomic regions (Rizzo and Buck, 2012; Hu et al., 2021) to increase sequencing depth in order to focus on biological regions implicated in human disorders. These approaches allow for identification of genetic mutations and epigenetic regulation that may underly psychiatric and neurological disorders [for more detailed reviews: (Rizzo and Buck, 2012; Shin et al., 2014; Shademan et al., 2021)].

RNA-Seq offers insight into differential gene expression (DGE), alternative splice variations, and novel transcript discovery. Subclasses of this technique include Whole Transcriptome Sequencing (WTS), mRNA Sequencing (e.g., poly-A enrichment), and small RNA sequencing (Hu et al., 2021). WTS involves sequencing the entire transcriptome, while mRNA and small RNA sequencing are enriched only for their respective RNA products. The initial workflow for RNA-Seq is similar to that of DNA-Seq, although RNA is generally converted into cDNA before amplification and sequencing (Chatterjee et al., 2018). Additionally, RNA-Seq analysis can be more challenging compared to DNA-Seq due to complexities accompanying the biological function and processing of RNA (i.e., alternative splicing, gene expression dynamics) (Li et al., 2014). There are over 100 variations of RNA-Seq being utilized today, many of which are derivations of two NGS strategies: short-read and long-read sequencing (Li et al., 2014). Direct RNA Sequencing (dRNA-Seq) further modifies the technique by circumventing cDNA synthesis steps and associated biases by directly sequencing the RNA template (Cartolano et al., 2016; Byrne et al., 2017; Garalde et al., 2018; Parker et al., 2020; Zhang et al., 2020).

Bioinformatic data analysis and management are essential for NGS, considering the incredible volume of data produced. A standard bioinformatics analysis workflow for RNA-Seq involves quality control assessment of raw data, read mapping and transcript assembly, quantification of mapped reads, and differential expression analysis between experimental samples, such as those related to human disease for RNA-Seq datasets (Li et al., 2014; Seqc/Maqc-Iii Consortium, 2014; Chatterjee et al., 2018). This general analysis workflow is typically divided into primary, secondary, and tertiary analysis. Primary analysis involves essential quality control measures such as read filtering, trimming, and quality scores. Secondary analysis encompasses read alignment to the genome of interest and variant calling (i.e., SNP and indel identification). Tertiary analysis then includes annotation and interpretation (e.g., functional annotation of novel variants) (Chatterjee et al., 2018). DNA-Seq and RNA-Seq frequently involve similar upfront analyses, however, their respective downstream bioinformatics pipelines will depend on the experimental hypothesis being tested [for more detailed reviews: (Dolled-Filhart et al., 2013; Klasberg et al., 2019; Pereira et al., 2020)].

The wide-ranging applications of NGS allow for it to be a useful tool in numerous fields, including molecular neuroscience. Sequencing technologies can allow insight into the underlying mechanisms of the healthy human brain and an array of brain-related disorders [for more detailed reviews: (Jiang et al., 2014; Shin et al., 2014; Wu et al., 2017a; Shademan et al., 2021; Sheardown et al., 2022)]. The myriad of applications for this technology has provided direct evidence of gene expression similarities between humans and mice demonstrating the translational value and potential limitations of animal models (Ray et al., 2018). WTS of allelic expression across healthy human brain regions has also furthered knowledge for genes harboring functional variants that can cause influence transcriptional regulation of RNA expression (Smith et al., 2013). Further, temporal changes in gene expression in response to specific events can be quantified, such as following spinal cord injury (Chen et al., 2013) and ischemic stroke (Bhattarai et al., 2017). NGS is also valuable for the study of neurodegenerative diseases, allowing for massive sequencing of DNA and RNA to identify novel mutations or dysregulation in transcriptomic patterns relevant to individual diseases (Shademan et al., 2021). NGS has produced various findings related to neuroscience and psychiatry (Table 1). Identification of alternative variants or susceptibility genes include: psychiatric risk gene *CACNA1C* (Clark et al., 2020), novel stroke-responsive lncRNAs (Bhattarai et al., 2017), Alzheimer’s-linked alternative splicing (Twine et al., 2011), schizophrenia-linked alternative promoter usage and splicing (Wu et al., 2012), rare immunological and neurodevelopmental mutations linked to obsessive compulsive disorder (Cappi et al., 2016), Tourette Disorder risk gene *PNKD* identification (Sun et al., 2018), and genes within specific molecular circuits linked to autism (Parikshak et al., 2013; Cotney et al., 2015). NGS expression information can then be further coupled with reverse genetics approaches in cell and animal models to study individual gene(s) of interest in greater detail (Twine et al., 2011; Wu et al., 2012; Cotney et al., 2015; Cappi et al., 2016; Bhattarai et al., 2017; Sun et al., 2018; Clark et al., 2020; Sinnamon et al., 2020).

**TABLE 1:** A selection of studies using NGS to study brain function and pathology.

Study	Species	Tissue	Disease/risk gene	Major finding
DeJesus-Hernandez et al., 2021	Human	Cerebellum	*C9orf72*	Pathological association discovery of *C9orf72* hexanucleotide expansion length
Clark et al., 2020	Human	Brain regions	*CACNA1C*	*CACNA1C* has a highly complex transcript profile
Hjelm et al., 2019	Human	Brain and blood	Mitochondrial	Identification and characterization of putative mitochondrial DNA deletions
Kim et al., 2016	Human	Hippocampus	Psychiatric	Differential activation of immune and inflammatory responses across disorders
Ramaker et al., 2017	Human	ACC, dlPFC, NAc	Psychiatric	Comprehensive transcriptome profiling across three disorders
Le et al., 2018	Human	Blood	Major depressive disorder	Identification and correlation of intramodular hub genes
Chen et al., 2013	Mouse	Spinal cord	Spinal cord injury	Characterization of temporal changes in gene expression after SCI
Sinnamon et al., 2020	Mouse	Hippocampus	Rett syndrome	Programmable RNA editing can repair mutations in mouse models
Bhattarai et al., 2017	Mouse	Cortex	Ischemic stroke	Ischemia induces distinct, temporal lncRNA expression changes. Identification of novel stroke-related lncRNAs
Farris et al., 2015	Human	Brain regions	Alcohol use disorder	Alcohol misuse produces alterations in transcriptome organization and transcripts
Ray et al., 2018	Human and mouse	Dorsal root ganglia	Chronic pain	Confirmation of murine model translatability to humans
Twine et al., 2011	Human	Brain lobes	Alzheimer’s disease	AD brain has differential gene expression and spice variants when compared to control
Wu et al., 2012	Human	Superior temporal gyrus	Schizophrenia	Schizophrenia STG has differential gene expression splice variants, and alternative promoter usage when compared to control
Zhou et al., 2011	Human	Hippocampus	Addiction	Shared and substance-specific changes in histones and gene expression
Akula et al., 2014	Human	dlPFC	Bipolar disorder	BD dlPFC has widespread transcriptome dysregulation when compared to control
Cappi et al., 2016	Human	Blood	Obsessive-compulsive disorder	Immunological, functional, and developmental gene enrichment largely in brain
Sun et al., 2018	Human	Blood	Tic disorders and Tourette syndrome	*PNKD* is associated with TD in a multiplex family
Cotney et al., 2015	Human and mouse	Fetal brain, hNSCs, embryonic cortex	Autism	Chromatin modifier CHD8 regulates autism risk genes during neurodevelopment
Parikshak et al., 2013	Human	Fetal and adult cortex	Autism	Molecular pathways and circuits found implicated in autism
Lorenzo et al., 2018	Human	Blood	Familial attention deficit hyperactivity disorder	Differential ADHD transcriptome expression and various pathway enrichments identified
Zeng et al., 2019	Human	Blood	Familial cortical myoclonic tremor with epilepsy	Pentanucleotide repeat expansion in *SAMD12* is the causative mutation

*ACC, anterior cingulate cortex; dlPFC, dorsolateral prefrontal cortex; NAc, nucleus accumbens; hNSC, human neural stem cell.*

### Next Generation Sequencing Strategies

#### Short-Read Second Generation Sequencing

Short-read sequencing is the standard method of detection and quantification of transcriptome-wide gene expression (Marioni et al., 2008; Mortazavi et al., 2008; Li et al., 2014; Seqc/Maqc-Iii Consortium, 2014; Chatterjee et al., 2018). It offers robust, large-scale comparisons of short DNA molecules ranging from 50–250 bp and produces tens of millions of reads in parallel (Stark et al., 2019; Hu et al., 2021). The two most popular second-generation sequencing platforms include Ion Torrent, which utilizes solid-phase amplification and emulsion PCR, and Illumina (Marioni et al., 2008).

Short-read sequencing is a high-throughput technique permitting millions of reads to be sequenced in parallel (Hu et al., 2021). Multiplexed sequencing allows for multiple libraries to be pooled and simultaneously sequenced, drastically reducing the price per sample as well as resolving issues associated with Sanger Sequencing (i.e., phasing issues and haploid versus diploid fragments) (Clarke et al., 2009; Hu et al., 2021). The reduction in cost and technological developments has led to a suite of computational tools and pipelines designed for short-read data analysis that make it more accessible to researchers from diverse fields (Li et al., 2014; Chatterjee et al., 2018). An additional advantage of short-read sequencing is the number of datasets that have already been produced and are readily available in public repositories (Iyer et al., 2015). Such independent datasets have been curated from tumors, healthy and diseased tissue, and cell lines, which can be used for *ab initio* assembly of short-read datasets. For example, the MiTranscriptome Project established that a large number of unannotated genes exist in the human transcriptome (i.e., long non-coding RNAs) that were not previously annotated by more traditional methodologies (Iyer et al., 2015).

Short-read RNA-seq technology unlocked a valuable toolbox for psychiatric and neurological research. It allowed for massively paralleled sequencing of various brain regions and tissues of control and diseased brains, thus identifying novel genes, and networks of genes, that are dysregulated in response to CNS disorders. For example, this method was utilized to compare the transcriptome profiles of various brain regions across healthy individuals and those who suffer from psychiatric disorders. Studies compared the anterior cingulate cortex, dorsolateral prefrontal cortex (dlPFC), nucleus accumbens (Ramaker et al., 2017) and hippocampus (Kim et al., 2016) of healthy controls and three psychiatric disorders: major depressive disorder (MDD), bipolar disorder, and schizophrenia. Transcriptome profiling for these disorders across multiple brain regions identified differentially expressed genes between the respective disorders when compared to control (Ramaker et al., 2017). In another study it was found that while the three above psychiatric disorders shared dysregulation of immune and inflammatory responses, the three presented differential activation when the profiles were compared to each other (Kim et al., 2016). RNA-Seq-based research into transcriptome dysregulation of psychiatric disorders provided insights into dlPFC for bipolar disorder (Akula et al., 2014), MDD-linked intramodular ‘hub’ genes (Le et al., 2018), superior temporal gyrus dysregulation in schizophrenia (Wu et al., 2012), and alternate pathway enrichments in familial attention deficit hyperactivity disorder (Lorenzo et al., 2018). Considering addiction as well, RNA-Seq of the hippocampus from individuals who suffered from cocaine- and alcohol-use disorder (AUD) were found to have extensive transcriptome dysregulation, both shared and substance-specific (Zhou et al., 2011). Additional research into specific brain regions of AUD individuals further cemented that alcohol misuse results in pronounced alterations in transcriptome expression that can differ across brain regions (Farris et al., 2015). Taken together, these studies highlight the differences found across the CNS, as well as in closely-linked human brain-related disorders.

Despite the advantages and the widespread use of short-read sequencing, a number of limitations still exist. These technologies generally have longer running times, short read bias, and an inability to identify novel transcript isoforms and structural variants (Whiteford et al., 2005). Due to the short reads produced by this technology, sequencing data generally has to be reassembled using a publicly accessible reference genome, which can result in structural challenges and the loss of important information on mRNA isoform frequency and splice variants. Novel isoforms can also be lost due to the improper realignment (Stark et al., 2019; Hu et al., 2021).

#### Long-Read Third Generation Sequencing

The development of long-read sequencing has overcome some of the challenges associated with second-generation sequencing. Long-read sequencing, also commonly referred to as third-generation sequencing, can directly sequence transcripts 10 kb or larger (Garalde et al., 2018). There are two main long-read technologies, Pacific Biosciences (PacBio) and Oxford Nanopore Technology (ONT) (Ip et al., 2015; Li et al., 2016; Byrne et al., 2017). While PacBio requires the conversion of RNA into tagged full-length cDNA transcripts, ONT can directly sequence either RNA or DNA, removing the need for cDNA synthesis and amplification (Stark et al., 2019). Direct RNA-Sequencing (dRNA-seq) is a derivative of long-read sequencing and does not require RNA fragmentation, cDNA synthesis, and PCR amplification because the RNA template is directly sequenced (Cartolano et al., 2016; Byrne et al., 2017; Garalde et al., 2018; Parker et al., 2020; Zhang et al., 2020). This removes biases associated with cDNA synthesis and amplification (Cartolano et al., 2016; Parekh et al., 2016; Byrne et al., 2017; Garalde et al., 2018), and allows for epitranscriptomic information to be retained, such as m^6^A modifications (Garalde et al., 2018; Parker et al., 2020; Zhang et al., 2020). Data analysis of long-read sequencing requires bioinformatics tools that can accurately assemble large reads, as ONT can generate up to 1 Mb reads and computationally stitch together >2 Mb reads (Miga et al., 2020).

One of the earliest studies that used long-read sequencing was conducted in birds (Korlach et al., 2017). This generated a more complete picture of the avian genome and provided further insight into gene structure, function, and evolution (Korlach et al., 2017). Using zebra finch and Anna’s hummingbird (two species widely studied in neuroscience) DNA as input for long-read sequencing via PacBio, produced reads in the megabase range with no gaps that resulted in a ∼150–200-fold improvement in their reference genomes, This resolution was previously unattainable, but was able to correct mis-assemblies, repeat structure errors, and sequence gaps that can further improve the use of these model organisms (Korlach et al., 2017).

Third-generation sequencing technology also provides an opportunity for advancing our understanding of human disorders. Long-read sequencing has the ability to discern information from complex genomic regions including repetitive elements, areas of high GC content, and detect more specific epitranscriptomic modifications not detectable by short-read NGS methods (Jain et al., 2018). Further, long-read sequencing can identify novel splice variants and profiles, which is invaluable to the study of psychiatric disorders where RNA splicing is suggested to be a key mechanism. One such example includes the psychiatric risk gene *CACNA1C*, which codes for the voltage-gated calcium channel Ca_*V*_1.2 (Clark et al., 2020). Long-read sequencing of the full-length coding transcripts provided evidence that the transcript profile is highly complex and the expression of alternatively spliced isoforms widely varies across brain regions that may be responsible for differential calcium channel function (Clark et al., 2020). Amplification-free long-read sequencing of the *C9orf72* gene in the cerebellum identified hexanucleotide expansion sizes, a frequent genetic cause of frontotemporal lobar degeneration and motor neuron disease, and uncovered significant correlation between expansion sizes and survival (DeJesus-Hernandez et al., 2021). Long-read NGS has also been used to identify a pentanucleotide repeat expansions within *SAMD12* as the causal mutation resulting in familial cortical myoclonic tremor with epilepsy (Zeng et al., 2019). These examples demonstrate the utility of long-read sequencing as an effective tool for the molecular basis of human disorders, especially for those diseases that cannot be discerned with more conventional NGS platforms (Zeng et al., 2019). While still in the early stages, long-read sequencing has already shown tremendous promise in unraveling the complexities of psychiatric and neurological disorders.

Advantages for long-read sequencing over short-read sequencing include the generation of extended reads for novel isoform identification, data collection in real-time offering faster turnaround, and its use for both DNA- and RNA-Seq (Clarke et al., 2009; Ip et al., 2015). Additionally, DNA amplification is not always required, avoiding the associated pitfalls (Parekh et al., 2016). However, long-read sequencing can result in a high rate of error due to signal-to-noise constraints (∼7–14%) when compared to short-read sequencing, although this error rate can decrease over time (Li et al., 2016). Additionally, long-read sequencing is reliant on high-quality RNA that contains full-length transcripts (Stark et al., 2019). Any sheering or degradation of the RNA during extraction may lead to 3′ bias, limiting usage for full-length transcriptomic analysis (Stark et al., 2019).

While price can be a disadvantage, ONT instruments are more cost-effective and smaller, allowing for more widespread use (Clarke et al., 2009; Li et al., 2016). Short-read sequencing may perform better for studies investigating differential gene expression due to its greater read depth, but long-read sequencing provides higher-resolution data for the study of complex psychiatric and neurological disorders. The decision on which NGS technique to employ should be based on the respective application, desired product, and the underlying hypothesis being tested. Both strategies are invaluable but offer their own set advantages and disadvantages.

### Single-Cell and Single-Nuclei RNA Sequencing

The brain is an extremely complex organ system with many unique regions and cell populations that coordinate molecular and behavioral phenotypes. Single-cell RNA sequencing (scRNA-Seq) has revolutionized transcriptomics by allowing for the characterization of individual cell populations that may be overlooked by bulk RNA-Seq (Tang et al., 2009), including their roles and responses in development, homeostasis, and disease (Darmanis et al., 2015; Zeisel et al., 2015; Tasic et al., 2016; Keren-Shaul et al., 2017; Jin et al., 2020). While the details vary with individual methods, scRNA-Seq functions similarly to bulk RNA sequencing. Cells are lysed and RNA is reverse transcribed to generate cDNA, which is then amplified and fragmented to generate NGS libraries that can be quantified (Tang et al., 2009). Unique oligonucleotide barcodes identify RNA from individual cells, and combinatorial indexing of barcodes can eliminate the need for cell-type specific isolation procedures (Rosenberg et al., 2018). scRNA-Seq methods are commonly either plate- or droplet-based. Plate-based methods use fluorescent-activated cell sorting (FACS) or microscope-guided capillary pipettes to place cells into wells, allowing for full-length transcript sequencing to distinguish between mRNA isoforms (Tasic et al., 2016; Paul et al., 2017). Droplet-based methods encase cells in individual lipid suspension droplets and mRNA is quantified by counting either the 5′ or 3′ ends (Macosko et al., 2015). These techniques can be combined with long-read sequencing tools such as ONT and hold potential for increasing transcript coverage and identifying more complex mRNA isoforms (Gupta et al., 2018).

scRNA-Seq has also been applied for powerful genetic screening of disease risk loci. Perturb-Seq is an approach that combines pooled CRISPR screening with scRNA-Seq and NGS analysis (Dixit et al., 2016; Schraivogel et al., 2020). This approach allows for high-throughput genetic screening that can dissect the results of genetic perturbations on the single-cell scale. Pooled screens allow for efficiency, while scRNA-Seq and NGS provide robust data on genetic and transcriptomic interactions (Dixit et al., 2016; Schraivogel et al., 2020). Combining the perturbation of multiple targets using CRISPR with scRNA-Seq has proven particularly useful in the study of complex diseases. Genome-wide association studies have identified a multitude of candidate genes for many psychiatric and neurological disorders, that would be cumbersome to individually screen. To understand the underlying genetics of autism spectrum disorder and neurodevelopmental delay (ASD/ND), Jin et al. (2020) were able to simultaneously introduce mutations in 35 ASD/ND risk genes in the mouse neocortex *in utero* and perform scRNA-Seq to assess how these changes impacted postnatal neuron and glial function. As most psychiatric and neurological disorders are due to a combination of genetic risk factors, the combination of CRISPR and RNA-Seq (i.e., Perturb-Seq) offers a unique capability to quickly screen multiple genetic perturbations, test physiological and behavioral phenotypes, and determine the biological activity of multiple candidate genes in a single study.

Single-nuclei RNA sequencing (snRNA-Seq) has emerged as a sister technique to scRNA-Seq (Grindberg et al., 2013). It can be used with samples incompatible with scRNA-seq, such as frozen samples and dense tissues. This makes snRNA-Seq particularly useful for neuroscience applications, as in addition to the density of brain tissue and the use of samples such as those frozen from postmortem human brains, the ramified processes of neurons and glial cells and their associated RNA are frequently lost in dissociated tissue. snRNA-Seq also may overcome some technical artifacts of scRNA-Seq, such as biases against specific cell types that are more likely to die during isolation (Tasic et al., 2016). snRNA-Seq is performed similarly to scRNA-Seq, following the same workflow and including similar well- and droplet-based protocols (Macosko et al., 2015; Tasic et al., 2016; Paul et al., 2017). Nuclear isolation can be performed using fluorescently-activated nuclei sorting (FANS), sucrose gradients, or other conventional nuclear extraction methods (Krishnaswami et al., 2016; Denisenko et al., 2020). snRNA-Seq also captures nuclear transcripts, including immature RNA molecules, allowing for unspliced RNA containing to be investigated (Bakken et al., 2018). It should be noted that the capture of nuclear transcripts can be both an advantage and a limitation depending on the experiment, as snRNA-Seq will not capture cytosolic RNA. However, using snRNA-Seq in conjunction with techniques such as laser-capture microdissection may allow for the determination of transcripts from other cellular regions such as dendrites to be analyzed from the same sample (Middleton et al., 2019).

scRNA-Seq and snRNA-Seq have proven invaluable for studying complex diseases by uncovering cell-type-specific vulnerability, protective mechanisms, and insights into pathogenesis. These approaches have been particularly useful for investigating complex and heterogenous disorders such as neurodegenerative diseases. Utilizing a transgenic mouse model for Alzheimer’s disease (AD) combined with scRNA-Seq observe glial-specific changes relevant to neuroinflammation and disease progression. This study identified a unique subset of microglia, termed disease-associated microglia (DAM), that has the potential to attenuate damage associated with AD. DAM were also confirmed in human AD postmortem brain tissue, as well as a transgenic mouse model of amyotrophic lateral sclerosis (ALS) (Keren-Shaul et al., 2017). As AD is a uniquely human disease, utilization of single-cell technologies in AD patients offers invaluable insight into the etiology of this disorder that can be further studied in cellular and animal models. One study performing snRNA-Seq in postmortem prefrontal cortex (PFC) of AD patients identified highly cell-type specific changes during various stages of the disorder, including genes that were perturbed in multiple cell types (Mathys et al., 2019). Combining single-cell and single-nuclei sequencing datasets across multiple organisms for the same disorder, such as AD, will allow for the development of more applicable and reliable animal models relevant to disease.

In addition to neurodegenerative disorders, snRNA-Seq has been employed to profile transcriptomic changes in the PFC of alcohol-dependent individuals. Similar to AD, differential expression analysis revealed that glial cells, including microglia, astrocytes, and oligodendrocytes, displayed the greatest change in gene expression in alcohol-dependent subjects. These cell-type specific observations corresponded with a significant enrichment of genes related to neuroinflammation, a phenomenon frequently associated with chronic alcohol misuse (Brenner et al., 2020). Multiple databases have been created that contain single-cell findings for specific diseases across laboratories and tissue sources (Cao et al., 2017; Jiang et al., 2020; Aging Atlas Consortium., 2021; Dong et al., 2021; Paisley and Liu, 2021; Zhao et al., 2021; Zheng et al., 2021). If utilized properly, these hold the promise of creating a single-cell molecular atlas of the brain in both healthy and diseased states.

scRNA-seq has proven invaluable for the identification of unique RNA expression between various cell types as well as differing responses to environmental contexts (Table 2), but it comes with important limitations. Multiple cells can be detected as a single cell in scRNA-Seq, forming doublets that can skew data and create false identifications. The use of FACS sorting as well as some computational methods including DoubletFinder and Scrublet can identify or reduce doublets (McGinnis et al., 2019; Wolock et al., 2019). In addition, many false negatives (commonly known as dropouts) result from incomplete RNA-recovery during tissue lysis, but other computational tools can impute or convert dropouts into a meaningful signal due to specific gene clusters displaying similar dropout patterns (Feng et al., 2020; Qiu, 2020; Zhang and Zhang, 2021). Non-physiological transcription from tissue dissection and cell dissociation can confound data, and dissociation and lysing removes important cellular and spatial context (Lacar et al., 2016; Wu et al., 2017b). Extra-physiological gene transcription occurs less in snRNA-Seq in comparison to scRNA-Seq. Challenges also exist in data analysis, where complex datasets are generated and computational methods and clustering procedures can be inconsistent. scRNA-Seq data are currently noisier than in bulk RNA-Seq, and biological variation between cell types can complicate analysis, but recently available tools can merge and more accurately align scRNA-Seq datasets across conditions, technologies used, and species (Butler et al., 2018). The single-cell transcriptomic field is still developing, though it holds tremendous promise to unlock a more comprehensive understanding of brain functioning as technologies continue to address these limitations. The standardization and transparency of single-cell data and computational methods will no doubt be an important facet of these improvements.

**TABLE 2:** A selection of studies using cell-type-specific transcriptomic methods to study brain pathology.

Study	Technique	Species	Tissue	Disease	Major finding
Jin et al., 2020	scRNA-Seq	Mouse	*In utero*, Neocortex	Autism spectrum disorders, neurodevelopmental delay	Cell type-specific gene modules in ASD risk genes
Keren-Shaul et al., 2017	scRNA-Seq smFISH	Human and Mouse	Whole Brain Hippocampus	Alzheimer’s disease, Amyotrophic lateral sclerosis	Novel microglia cell type associated with AD and ALS
Mathys et al., 2019	snRNA-Seq	Human	PFC	Alzheimer’s disease	Cell type-specific expression changes during disorder progression, consistent changes in myelination-related genes.
Brenner et al., 2020	snRNA-Seq	Human	PFC	Alcohol dependence	Greatest change in gene expression within glial cells; significant enrichment of neuroinflammation-related genes
Zheng et al., 2022	scRNA-seq	Mouse	Whole brain hemispheres	Ischemic stroke	Cell type-specific transcriptional changes with neuroinflammation
Liu et al., 2020	scRNA-Seq	Mouse	Brainstem	Amyotrophic lateral sclerosis	Cell-type specific transcriptional changes in multiple ALS-related pathways
Nguyen et al., 2020	snRNA-Seq	Human	Middle frontal neocortex	Alzheimer’s disease	Identified depleted microglia subpopulations in AD patients with APOE and TREM2 risk variants
Sadick et al., 2022	snRNA-Seq	Human	PFC	Alzheimer’s disease	Global and subtype-specific transcriptomic changes in glia
Wertz et al., 2020	snRNA-Seq	Mouse	Striatum	Huntington’s disease	Loss of IL-6 augments behavioral phenotypes and mutant huntingtin dysregulation of genes related to HD pathology
Zhong et al., 2020	snRNA-Seq	Mouse	Hippocampus	Alzheimer’s disease	Neuronal subtype-specific and disease progression-related transcriptional changes
Schirmer et al., 2019	snRNA-Seq	Human	Cortical gray matter and subcortical white matter; lesions	Multiple sclerosis	Cell type-specific changes in gene expression in MS lesions and surroundings

*PFC, prefrontal cortex.*

### Epigenomic and Translatomic Techniques

While bulk RNA sequencing can provide a plethora of data about the transcriptome, more specialized techniques have been developed to delve into the epigenome and translatome of the brain. Translating ribosome affinity purification sequencing (TRAP-Seq) involves cell-type-specific, indirect tagging of mRNAs through targeted transgene expression of an affinity tag (such as enhanced green fluorescent protein) associated with the large ribosomal subunit protein L10a (Heiman et al., 2014). The affinity tag simultaneously allows for visualization of the cell type of interest within tissue as well as purification of translated mRNA. Purified mRNA can then be analyzed downstream, including with RNA-Seq. A notable advantage of this technique is that it does not require dissociation of cells within tissue or mRNA capture from specific cell types (Heiman et al., 2014). Another method known as ribosome profiling identifies the exact mRNA fragments currently being translated by ribosomes (McGlincy and Ingolia, 2017). Nuclease digest fragments of all sequences not enclosed within ribosomes, and a cDNA library is generated from the remaining RNA, which then undergoes high-throughput sequencing (McGlincy and Ingolia, 2017). Ribosome profiling thus provides a snapshot of ribosome locations on the transcriptome and can provide high-resolution information on translational perturbations, such as stalling, as a result of experimental perturbations (McGlincy and Ingolia, 2017). Ribosome profiling is possible at the single cell level (VanInsberghe et al., 2021), and has been modified to only isolate active ribosomes, addressing a prior limitation of the technique (Clamer et al., 2018). Both TRAP-Seq and ribosome profiling have proven useful in studying neurologic disease. Several studies have recently employed TRAP-Seq to study cell-type-specific changes in gene expression and translation in neurodegenerative diseases such as AD (McKeever et al., 2017; Hinman et al., 2021), and translational repression in cortical neurons of Rett syndrome patients (Rodrigues et al., 2020). Ribosome profiling has revealed changes in ribosomal occupancy of genes as well as ribosome stalling in Huntington Disease (HD) models (Sharma et al., 2020; Eshraghi et al., 2021), and has similarly illuminated cell-type-specific alterations in the translatome in gliomas, AD, Parkinson’s disease, epilepsy, prion disease, and X-linked intellectual disability (Gonzalez et al., 2014; Kim et al., 2019b, 2021a; Scheckel et al., 2020; Nagayoshi et al., 2021; Eastman et al., 2022).

As epigenetic modifications were recognized to play a critical role in brain function and behavior, sequencing techniques to examine epigenetic modifications to the transcriptome were developed. In 2007 chromatin immunoprecipitation with massively parallel sequencing (ChIP-Seq) emerged as a method that permitted the identification of binding sites for DNA-associated proteins such as transcription factors and histones (Robertson et al., 2007). While ChIP-Seq opened the door for numerous epigenetic studies, applying ChIP-Seq to single-cell studies has been challenging due to limited material recovery. Recent efforts have combined other methods with ChIP-Seq to improve yield, including the use of DNA barcoding/indexing and microfluidics, but data from individual cells remains sparse, and more sensitive adaptations involve tedious library preparation processes (Robertson et al., 2007; Mundade et al., 2014; Rotem et al., 2015; Ai et al., 2019). As an alternative to ChIP-Seq, Cleavage Under Targets and Release Using Nuclease (CUT&RUN) was developed to overcome some of the challenges associated with other epigenomic profiling methods (Skene and Henikoff, 2017). Based on the chromatin immunocleavage method (Schmid et al., 2004), an MNase is tethered to the DNA using transcription factor specific antibodies, allowing for precise cutting of DNA at transcription factor binding sites. The cut fragments of DNA are then be used for library preparation and sequenced, allowing for study of transcriptionally active sites of the genome (Skene and Henikoff, 2017). Due to a reduction in background levels, CUT&RUN requires fewer reads, lowering the cost for sequencing and making it a more accessible technique (Skene and Henikoff, 2017). Recently, single cell CUT&RUN has been developed to examine the epitranscriptomic landscape of individual cells (Hainer et al., 2019; Yu et al., 2021).

In addition, Assay for Transposase-Accessible Chromatin with high-throughput sequencing (ATAC-Seq) has been used in multiple contexts to observe the epigenome in both bulk and single-cell sequencing studies (Buenrostro et al., 2013; Cusanovich et al., 2015). In ATAC-Seq, chromatin are incubated with the Tn5 transposase which is complexed with adapter sequences, taking advantage of this enzyme’s hyperactivity in fragmenting accessible DNA (Marinov and Shipony, 2021). By using the Tn5 transposase, ATAC-Seq simultaneously fragments DNA and inserts oligo sequencing adapters that can be utilized for both NGS and PCR (Buenrostro et al., 2013). ATAC-Seq has also been adapted for single-cell CRISPR screening (Perturb-ATAC) to elucidate gene regulatory networks (Rubin et al., 2019), which could become a powerful tool for studying the epigenetic regulation of development and disease.

Along with RNA-Seq methods, ATAC-Seq can help uncover transcription factor effects on gene expression of particular transcripts. One study employing ATAC-Seq in addition to ChIP-Seq identified non-coding regulatory regions (including promotors and proximal/distal enhancers) that drive the transcriptomic identity of all major cell types in human brain, as well as their relative roles in AD and multiple human psychiatric disorders (Nott et al., 2019). Another study performed single-cell ATAC-Seq (scATAC-Seq) to uncover epigenetic variation in AD and Parkinson’s disease (Corces et al., 2020). DNA methylation assays are also possible in single-cells utilizing plate-based nuclei sorting, bisulfite conversion, and multiplexed sequencing, though these must be developed to be more efficient as they lack extensive coverage (Luo et al., 2017). Droplet-based methods could assist with this, but whole-genome sequencing remains cost-prohibitive for assessing the epigenome of individual cell-types. Technologies are rapidly developing toward single-cell multiomics, allowing for the characterization of discrete cell types at virtually all fundamental levels of cellular identity and functioning (Welch et al., 2019; Zhu et al., 2021). Table 3 highlights a number of studies that employ the discussed epigenomic and translatomic techniques. While still in development, many of these techniques have been combined with scRNA-seq and will undoubtedly play a valuable role in understanding the cell-type specific contribution to human disorders.

**TABLE 3:** A selection of studies using translatomic and epigenomic methods to study brain function and pathology.

Study	Technique	Species	Tissue	Disease	Major finding
Nott et al., 2019	ChIP-Seq ATAC-Seq	Human	Resected cortical brain tissue	Alzheimer’s disease, Psychiatric disorders	Cell type specificity of non-coding regulatory elements
Corces et al., 2020	scATAC-Seq	Human	Multiple brain regions	Alzheimer’s disease, Parkinson’s Disease	Characterized inherited non-coding epigenetic variation in AD and PD
Wang et al., 2021	ATAC-Seq	Human	Tumor tissue	Pediatric high-grade glioma	Tumor-specific oncogene enhancers/regulatory networks and potential genomic structural changes
Li et al., 2020	ChIP-Seq scRNA-Seq	Human	Brain, neuronal cells	Major depressive disorder	Binding disruption of 15 transcription factors at 34 MDD risk SNPs
Kouakou et al., 2021	ATAC-Seq	Human	Fetal PFC	Multiple psychiatric disorders	Significantly enriched heritability for SNPs associated with psychiatric disorders within open chromatin regions
Bryois et al., 2018	ATAC-Seq	Human	PFC	Schizophrenia	Enrichment of schizophrenia SNP heritability in accessible chromatin regions
Eshraghi et al., 2021	Ribosome profiling, RNA-Seq	Human and Mouse	Striatal neurons Fibroblasts	Huntington’s disease	Mutant huntingtin results in ribosome stalling in multiple identified genes
Kim et al., 2021a	Ribosome profiling	Mouse	Striatum, ventral midbrain	Parkinson’s disease	Preferential mRNA translation with G2019S LRRK2 mutation
McKeever et al., 2017	TRAP-Seq	Mouse	Anterior forebrain cholinergic neurons and their cortical/hippocampal projections	Alzheimer’s disease	Differential gene expression in cholinergic neurons of TgCRND8 mice
Hinman et al., 2021	TRAP-Seq	Mouse	Central Nervous System	Tauopathy	miR-142-3p regulation of gene expression networks in oligodendrocytes is involved in neurodegeneration
Jiwaji et al., 2022	TRAP-Seq	Human and Mouse	Astrocyte culture	Alzheimer’s disease	Astrocyte Aß and Tau pathology are distinct, but overlapping in gene expression profiles
Kim et al., 2019b	Ribosome profiling	Mouse NIH/3T3 cells	Cortical neurons	Intractable epilepsy	Identified translational dysregulation mechanism in *MTOR* mutations
Eastman et al., 2022	Ribosome profiling	Mouse	Cerebral cortex	Alzheimer’s disease	AD mouse model differences in translatome regulation
Simard et al., 2018	TRAP-Seq	Mouse	Cortical astroglia	Chronic stress, depression, anxiety	Decreased expression of astroglial plasticity genes
Sharma et al., 2020	Ribosome profiling	Mouse	Striatal cells	Huntington’s disease	Altered LC3A and LC3B ribosomal occupancy

*PFC, prefrontal cortex.*

### Spatially-Resolved Transcriptomics

One of the fundamental limitations of scRNA-Seq is the loss of spatial/tissue context, which is critical in cell identity and function. Spatially-resolved transcriptomics overcomes this by adding spatial resolution to RNA sequencing at the nearly single-cell level. Spatial information has been added to single-cell datasets through laser-capture microdissection, such as in geographical position sequencing (Geo-Seq) (Chen et al., 2017). Other common spatially-resolved techniques can be classified into three major categories: fluorescent *in situ* hybridization (FISH)-based, *in situ* sequencing, and *in situ* capturing (Noel et al., 2021; Ortiz et al., 2021; Rao et al., 2021).

Two of the leading FISH-based methods include single-molecule fluorescence *in situ* hybridization (smFISH) and its multiplexed form, MERFISH (multiplexed error-robust fluorescence *in situ* hybridization) (Femino et al., 1998; Xia et al., 2019). smFISH uses oligonucleotide probes and is capable of detecting the distribution of single RNA molecules with high resolution (Femino et al., 1998; Raj et al., 2008). This not only allows for the identification of spatial information within a tissue, but smFISH can also reveal subcellular localization of RNA. While smFISH is capable of this subcellular localization, assigning transcripts to individual cells can be difficult in dense brain tissue with highly-ramified cell processes, though computational image analysis tools are improving to mitigate this issue (Maynard et al., 2020). In addition, FISH-based methods require probes for predefined targets, and can be complex and time-consuming to employ, meaning only smaller tissue volumes can be adequately analyzed. Newer techniques are being developed to increase smFISH efficiency, particularly in dense tissues (Sun et al., 2019).

*In situ* sequencing methods such as FISSEQ accomplish RNA sequencing on tissue samples; cDNA synthesis is accomplished via padlock probes or stably cross-linked cDNA amplicons (Lee et al., 2014, 2015). Rolling-circle amplification is used, and samples are then sequenced, removing the need for RNA extraction. One advantage of this approach over FISH-based methods is the ability to visualize unknown RNA sequences, though both FISH-based and *in situ* sequencing methods require tissue to be imaged and then processed down to the single-cell level through segmentation (Rao et al., 2021).

*In situ* capturing methods comprise a diverse range of spatially-resolved transcriptomic methods in which pre-patterned slides with oligonucleotide barcodes or barcoded beads are used to identify the location of origin for a given RNA, which is then sequenced outside of the tissue using single-cell transcriptomic methods (Ståhl et al., 2016; Rodriques et al., 2019). *In situ* capturing includes the method commonly referred to as Spatial Transcriptomics (ST or Visium) as well as Slide-Seq, and many of these methods are NGS-based (Rodriques et al., 2019; Stickels et al., 2021). *In situ* capturing methods of spatially-resolved sequencing allow for higher throughput, as greater tissue areas can be covered with less time and resources than imaging-based methods (Noel et al., 2021). Tissue is histologically stained (commonly with hematoxylin and eosin) and imaged to map spot locations relative to known landmarks (Longo et al., 2021; Noel et al., 2021). cDNA synthesis is performed *in situ* and resulting cDNA-RNA hybrids are cleaved off slides (Rodriques et al., 2019; Stickels et al., 2021). The barcodes can identify RNA in resulting sequencing libraries. Newer methods such as Slide-Seq come closer to single-cell resolution (possessing a spatial resolution of ∼10 μM) but have reduced detection efficiency (Rodriques et al., 2019; Stickels et al., 2021).

Spatially-resolved transcriptomics can aid in our understanding of complex neural circuits underlying behavior in both the healthy and diseased brain (Table 4). In combination with scRNA-Seq, MERFISH uncovered distinct divisions of the mouse hypothalamus responsible for varying social behaviors (Moffitt et al., 2018; Kim et al., 2019a). smFISH was utilized in the study identifying DAM to localize these novel microglia near Aβ plaques (Keren-Shaul et al., 2017). Slide-Seq was originally used to map cell types in mouse cerebellum and hippocampus, as well as characterize cortical cell type-specific responses in a model of traumatic brain injury (Rodriques et al., 2019). Spatially-resolved transcriptomics has also proven valuable in studying neurodegenerative diseases, where the roles of neuropathological hallmarks are still being investigated (Keren-Shaul et al., 2017; Maniatis et al., 2019; Chen et al., 2020; Gregory et al., 2020; Navarro et al., 2020). One recent study used spatial transcriptomics and *in situ* sequencing methods in an AD mouse model to assess transcriptional changes in cells surrounding Aβ plaques and found significant changes in discrete gene co-expression networks in the early and late stages of the disease (Chen et al., 2020). Spatially-resolved transcriptomics holds tremendous promise for revolutionizing our knowledge of brain region specific functions in both a healthy brain and in pathological contexts.

**TABLE 4:** A selection of studies using spatially-resolved transcriptomic methods to study brain function and pathology.

Study	Technique	Species	Tissue	Disease	Major finding
Keren-Shaul et al., 2017	scRNA-Seq smFISH	Human and Mouse	Whole brain Hippocampus	Alzheimer’s disease, Amyotrophic lateral sclerosis	Novel microglia cell type (DAM) localized near Aβ plaques
Rodriques et al., 2019	Slide-seq (*in situ* capturing)	Mouse	Cortex	Traumatic brain injury	Cell type-specific effects during stages of traumatic brain injury
Chen et al., 2020	Spatial transcriptomics (*in situ* capturing), *in situ* sequencing	Human and Mouse	Coronal sections. Superior frontal gyrus	Alzheimer’s disease	Identified gene co-expression networks related to Aβ plaque formation
Navarro et al., 2020	Spatial transcriptomics (*in situ* capturing)	Mouse	Brain	Alzheimer’s disease	Spatial mapping of differentially-expressed genes in AD
Willis et al., 2020	Spatial transcriptomics (*in situ* capturing)	Mouse	Brain	Traumatic Brain Injury	Repopulation of microglia positively affects injury microenvironment
Hasel et al., 2021	Spatial transcriptomics (Visium, *in situ* capturing)	Mouse	Cortical astrocytes	Neuroinflammation	Localization of reactive astrocyte substates
Kaufmann et al., 2021	Spatial transcriptomics (*in situ* capturing)	Human	Brain	Multiple sclerosis	Identify and localize T cell population in MS brains
Gregory et al., 2020	Spatial transcriptomics (Visium, *in situ* capturing)	Human	Brain	Amyotrophic lateral sclerosis	*GRM3* and *USP47* transcripts spatially dysregulated
Kiss et al., 2022	Spatial transcriptomics (Visium, *in situ* capturing)	Mouse	Brain	Aging	Senescent cell microdomains identified in multiple brain regions based on gene expression
Maniatis et al., 2019	Spatial Transcriptomics (*in situ* capturing)	Human and Mouse	Spinal cord	Amyotrophic lateral sclerosis	Established a time course of molecular mechanisms of disease progression

Through unbiased exploration of molecular tissue organization and gene expression, spatially-resolved transcriptomics has the potential to redefine neuroanatomy (Close et al., 2021; Ortiz et al., 2021). However, similar to scRNA-Seq, spatially-resolved transcriptomics generates large datasets, is prone to technical bias and noise, and assumes a one-to-one relationship between spots and cells. While an individual spot may be roughly the size of a single cell, it may overlap multiple cells, especially with the highly-branched nature of neurons and glia (Cardona-Alberich et al., 2021; Close et al., 2021). As technology develops, scalability and resolution will increase (Rao et al., 2021). The gene expression data captured by spatially-resolved transcriptomics lacks the transcriptomic breadth of scRNA-Seq, and thus their combination produces powerful information on cell identity and interactions (Longo et al., 2021). While spatially-resolved transcriptomics reveals rough gene expression “neighborhoods” in tissue regions, scRNA-Seq provides more detailed information. Especially when combined with scRNA-Seq, spatially-resolved transcriptomics can be used to understand tissue- specific dynamics of development, dysregulation and disorganization in disease, and responses to the environment and perturbations (Longo et al., 2021; Rao et al., 2021). Thus, a comprehensive understanding of molecular identity and functioning can be achieved in model organisms.

## Advances in the Analysis of Behavioral Neuroscience

### Translation of Animal Behavior to Humans

Observing animal behavior in the context of their environment and biology is fundamental to understanding corresponding phenotypes (Hogan et al., 2009; Nestler and Hyman, 2010; Tinbergen, 2010; Stewart and Kalueff, 2015; Gomez-Marin and Ghazanfar, 2019). The lack of tools to rigorously analyze organismal behavior has left a disconnect between molecular neuroscience and behavioral phenotypes. Traditionally, recordings of animal behavior have been analyzed by trained researchers’ post-experimentation, but analyses of these videos suffer from limitations including experimenter bias and differences in training. Current methods of interpretation and analysis of animal behavior remain subjective, limiting translation to human behavior and disease. To circumvent these issues, researchers have recently begun to integrate computational and machine learning approaches with neurobehavioral assessments (Poggio, 1981; Krakauer et al., 2017). Through this integrative approach, more accurate animal models can be generated for an array of human conditions with strong validity and high reproducibility (van der Staay et al., 2009).

Animal models of psychiatric and neurological disorders are often regarded as poor predictors for human disorders, limiting the development and testing of novel treatments. For example, ALS is a fatal neurodevelopmental condition with few effective treatment options (Meshalkina et al., 2017). In recent years, several medications have shown promise in treating mouse models of this disorder, but have subsequently failed in human clinical trials (Perrin, 2014). Investigation into this discrepancy revealed that the progression of motor degeneration exhibited by mutant mice with a dysfunction in *TDP43*, the gene encoding the TDP43 protein responsible for the harmful protein aggregates associated with ALS (Brettschneider et al., 2013), is subtle and fast. Successful treatment of ALS in clinical trials relies on how well experimenters can implement multivariate levels of analysis to characterize early behavioral changes in an animal model (Perrin, 2014; Meshalkina et al., 2017).

In addition to the creation of genetically modified animals using CRISPR and other strategies, researchers have used selective breeding to isolate heritable traits relevant to human disorders. For example, selective breeding of alcohol-preferring and apomorphine susceptible rats was undertaken to model AUD and schizophrenia, respectively (Colombo et al., 1995; Ellenbroek and Cools, 2002). Both phenotypes in these instances are predisposed by genetic factors and can benefit from a deeper level of behavioral analysis. In depth analysis of behavioral traits using machine learning can assist this approach by identifying the most behaviorally relevant animals before beginning the laborious task of breeding multiple generations of animals.

Traditional behavioral analyses are now being augmented with modern computational approaches to improve our understanding of behavioral outcomes, advance the field of molecular neuroscience, and discern causal factors of human disorders. Deep learning allows the transformation of raw data into discrete representations of diverse behavioral domains. Such transformations allow for complex tasks to train machine learning algorithms and eventually make predictions of behavior. Deep learning has previously been used across multiple scientific domains, including screening of drug targets, creating brain networks, and predicting mutations from noncoding DNA (Ciodaro et al., 2012; Helmstaedter et al., 2013; LeCun et al., 2015; Xiong et al., 2015). Herein we will examine the recent developments in using deep learning to analyze animal behavior relevant to modeling human conditions.

### Deep Learning for Automated Analyses of Behavior

Computational analysis can aid in understanding dynamic animal behavior influenced by genetic and environmental factors. However, not all computational analysis techniques are well suited to behavioral neuroscience studies. Supervised learning models must first analyze enormous sets of data for algorithms to predict future circumstances, which are not readily available to the majority of neuroscience laboratories (LeCun et al., 2015). Traditional animal posture tracking relies on physical markers, skeleton contour models, and training regressors based on derived features from animals. These methods have multiple inherent limitations which can hinder research efforts. In contrast, deep learning leads to the development of multifaceted and complex neural networks that are robust enough to predict behavior with limited training data (Mathis and Mathis, 2020). Multiple platforms such as MoSeq, DeepHL, DeepPoseKit, SLEAP, and DeepLabCut utilize deep learning approaches for pose estimation of animal behaviors. DeepLabCut is one of the most widely used deep learning platforms for behavioral analysis, overcomes many limitations of animal posture, and offers reliable tracking of behavior across different environments (Mathis et al., 2018).

DeepLabCut is an open-source toolbox that provides reliable and efficient tools for high throughput video analysis (Mathis et al., 2018; Nath et al., 2019) which incorporates features from DeeperCut and ImageNet, a pose estimation algorithm previously used in humans and for object recognition. Video recordings of animal behavior are obtained then labeled and dissected within the DeepLabCut toolbox. An automated learning module extracts frames that best represent the observed animal behavior and the user individually labels points of interest on the animals’ bodies in those extracted frames to create a stable training dataset. After achieving sufficient reliability across multiple animals and experiments, the network of data (ResNet) is capable of automating the entire process to predict the desired labels in future video recordings (Mathis et al., 2018; Nath et al., 2019). DeepLabCut is extremely versatile, permitting the user to continually refine the labeling parameters to make feature detection more accurate and detect variable behaviors.

DeepLabCut has several advantages and disadvantages over traditional behavior analyses. Providing a user-friendly interface, DeepLabCut can achieve human-level accuracy on analyzing behavior (Nath et al., 2019). Additionally, utilizing the automated analysis minimizes the cost of manually analyzing video recordings of behavior. DeepLabCut, however, can require extensive processing power not universally available. Another limitation DeepLabCut shares with other tracking software is that some body features of the subject may be occluded in the video and will not be tracked. To address this limitation, DeepLabCut predicts the outcome of occluded body parts and quickly detects the labeled body part once they reemerge in-frame (Nath et al., 2019). DeepLabCut can also be used to track multiple different animals at once, including in their home environments (Lauer et al., 2021). The majority of model organisms used in neuroscience research, such as rodents and non-human primates, are inherently social animals. Group housing permits more naturalistic expressions of behavior, as well as the observation of social and hierarchical dynamics.

DeepLabCut has been used to characterize an array of phenotypes across a number of different species. For example, to understand the role of CYP20A1 in development and neurological function, a recent study generated a mutant *cyp20a1-/-* zebrafish (Brun et al., 2021). Utilizing DeepLabCut for the automated analysis of swimming behavior in a novel tank assay demonstrated that the CYP20AI locus plays a critical role in regulating cortisol and anxiety-like behavior (Brun et al., 2021). In a separate example, Chen et al. (2021) generated a knockout mouse of the transcriptional factor MYT1L to study its role in neurodevelopment and behavior. Humans with a mutation in MYT1L display a hyperactive phenotype. Postural analysis of mice using DeepLabCut during an open field paradigm found sex dependent effects on activity which, along with other findings, validated this mouse as an appropriate model for the human disorder (Chen et al., 2021). DeepLabCut, as well as other similar tools, is thus a powerful tool to analyze multiple the for specific phenotypes related to the development of human disease.

While DeepLabCut is mentioned extensively in this review, the existence of similar but separate algorithms exists, such as DeepHL, DeepPoseKit, and LEAP. DeepHL is similar to DeepLabCut in that it uses deep neural networks for automated analysis of behavior. Maekawa et al. (2020) developed a matrix capable of classifying trajectories across multiple species. However, data obtained from a deep neural network is frequently considered a black box due to the difficulty in the interpretation of high–level features. DeepHL can illuminate characteristic segments in the trajectories of a gross deep learning data set to detect group differences, one example being flight patterns of male and female seabirds (Maekawa et al., 2020). DeepPoseKit offers an advanced pose estimation kit which utilizes an enhanced deep learning neural network called Stacked DenseNet to provide pixel – accurate tracking of body parts of an animal as opposed to DeepLabCut’s convolutional neural network (Graving et al., 2019). LEAP combines a graphical user interface for the labeling of images with a simple network architecture that allows the algorithm to generate predictive animal behavior with as little as ten frames of data, and can now be used to track multiple animals at once (Pereira et al., 2019, 2022). These types of advancements in computer science have opened the door for a wide variety of deep learning algorithms to be implemented in the field of behavioral analysis.

### Additional Computational Advances in Behavioral and Genomic Analysis

The progression of behavioral analysis technology has not kept pace with the remarkable development of neurocircuit manipulations (Anderson and Perona, 2014; Datta et al., 2019) and high-throughput sequencing. DeepLabCut is just one of many examples that utilizes deep learning algorithms for the analysis of behavior. Research in the pain field utilizing computational techniques has demonstrated that meaningful data can be obtained from small changes in temporal resolution of stimulus evoked behavior (Fried et al., 2020). Using deep learning algorithms, an automated pain assessment platform (Pain Assessment at Withdrawal Speeds; PAWS) was developed which automatically scores behavioral features seen in the withdrawal reflex (Jones et al., 2020). PAWS uses distinct behavioral features to create a single pain score that is capable of identifying a unique paw withdrawal reflexes between different genetic strains of mice (Jones et al., 2020).

Drosophila exhibit a tremendous diversity of behavior and can serve as an animal model of human neurodegenerative diseases. Motor movement of flies has been extensively studied using optical touch sensors that track movement as the fly comes into contact with the sensor (Mendes et al., 2013). However, using this method only utilizes one variable of fly locomotion and leaves out microscale leg movements. Wu et al. (2019) developed a machine learning algorithm to automatically track leg movements of freely moving flies called Feature Learning-based Limb segmentation and Tracking (FLLIT). FLLIT automatically tracks drosophila leg movement and generates a series of gait parameters with minimal training input. FLLIT accurately tracks disrupted gait in Parkinson’s Disease and Spinocerebellar ataxia 3 mutant flies, indicating that these genetic models captured motor deficits frequently observed in patients (Wu et al., 2019).

Non-human primates are one of the most important model organisms for understanding human disorders. Unfortunately, only a fraction of the available data generated by these complex organisms are typically used. DeepLabCut and other similar tools may have trouble tracking non-human primates due to their large range of movements and the lack of a database of systematically defined behaviors. OpenMonkeyStudio was developed to specifically track non-human primate motion using their own database of annotated macaque images and pose detection software (Bala et al., 2020). OpenMonkeyStudio can provide laboratories with the necessary toolbox to track and analyze complex behaviors exhibited by rhesus macaques, and potentially other non-human primates. The authors note that their system is not a simple plug and play solution to understand non-human primate behaviors, and OpenMonkeyStudio will need to be replicated in each individual laboratory before data collection can occur (Bala et al., 2020). Nevertheless, this is the first instance of deep learning technology specifically optimized for tracking rhesus macaque monkeys.

Behavioral data is most reliable when collected in the animal’s natural environment with minimized human intervention. The deep learning algorithm You Only Look Once (YOLO) accurately and reliably captures feeding behavior in farm raised pigs. Experimenters captured recordings of group housed, farm-raised pigs using YOLO to determine differences in food and water intake (Kim et al., 2021b). YOLO may assisted in real-time monitoring of changes in feeding and drinking behavior that are a result of physiological differences between animals or subtle changes in the environment. By adding multiple different detection layers to the YOLO algorithm, the system can accurately track multiple overlapping pigs within a designated housing area (Kim et al., 2021b). Pigs are increasingly being used as models of psychiatric disorders and other human diseases (Gieling et al., 2011), suggesting that YOLO may be a valuable deep learning tool for understanding human behavior.

Using deep learning to analyze genotype and behavior concurrently can derive an accurate organismal phenotype. Geneticists can trace quantitative trait loci to specific phenotypes to establish a causal genotype-to–phenotype relationships (Jansen and Nap, 2001). Geneticists are now using deep learning approaches to efficiently identify quantitative trait loci and single nucleotide polymorphisms for thousands of emergent phenotypes, both physiological and behavioral. For example, DeepSVP is a deep learning algorithm that leverages genomic information obtained in ontologies and phenotypes obtained in mouse loss-of-function studies to accurately predict whether a structural variant in the human genome will be pathogenic (Althagafi et al., 2021). Table 5 summarizes different, novel deep learning methods that are being used to characterize phenotypes based on genetics and morphology, in addition to behavior. As the integration of genomics and phenotype characterization using deep learning is very new, studies outside of the field of neuroscience have also been highlighted.

**TABLE 5:** A selection of studies using deep learning to integrate genomic sequencing and behavioral phenotyping.

Study	Deep learning method	Subject	Trait/pathology analyzed	Major finding
Gurovich et al., 2019	DeepGestalt	Human morphology	Angelmen’s, Noonan, Cornelia de Lange Syndrome	Achieved over 90% accuracy in detecting the prevalence of a syndrome based facial features
van Hilten et al., 2021	GenNet	Human genomics	Schizophrenia	Identified and predicted the phenotypic outcome at a rate of 0.685 (AUC)
Wang et al., 2019	WheatNet	Wheat morphology	Flowering time	Determined accurate predictions of morphology controlled by genetics
Cheng et al., 2021	XGBoost	Arabidopsis and maize genomics	Nitrogen use efficiency (NUE)	Predicted important genes in both species that aid in maximal NUE
Sheehan and Korstanje, 2018	Llastik	Kidney glomeruli morphology	Renal function	Provided fast and accurate glomerular identification and features
Jones et al., 2020	PAWS	Mouse behavior	Affective pain	Detects mechanical hypersensitivity that otherwise would not be determined using von Frey
Wu et al., 2019	FLLIT	*Drosophila* behavior	Neurodegeneration	Recapitulated gait characteristics of human neurodegenerative diseases using models of Parkinson’s and mutant SCA3 flies
Bala et al., 2020	OpenMonkey Studio	Monkey behavior	Pose estimation in a social setting	Describes a deep learning approach for tracking freely moving macaques in an unconstrained environment
Kim et al., 2021b	YOLO	Weanling pig behavior	Feeding	Detects feeding locations from non – feeding locations and detects feeding behavior with precise accuracy to classify pig age
Abdollahi-Arpanahi et al., 2020	MLP and CNN’s	Holstein bull genomics	Sire conception rate (SCR)	Correlated SCR traits in bulls based on non – additive loci
Althagafi et al., 2021	DeepSVP	Human and mouse genomics	Dravet Syndrome	Assess genotype in humans using genomic and phenomic data in loss – of – function mouse studies

## Conclusion

The development of novel techniques to study a diverse array of model organisms using multiple species has been at the forefront of neurobiological research. With the advent of CRISPR genome editing, it is now easier than ever for independent laboratories to design and create their own experimental animals. Genomic and behavioral neuroscience technologies have also advanced in parallel to genome engineering. To circumvent some of the issues present in second-generation short-read sequencing, third-generation long-read sequencing allows for the production of longer transcripts necessary for novel isoform discovery. scRNA-Seq and snRNA-Seq now allow researchers to understand changes in gene expression on the single cell level, which is particularly important when studying an organ with such cellular heterogeneity as the brain. Combined with spatial transcriptomics, studies can now investigate brain region and cell type changes in model organisms. Beyond genomics, the integration of deep learning algorithms into behavioral analysis software allows for quicker and more in-depth analysis of behavioral phenotypes.

The exponential rise in genetic, genomic, and behavioral neuroscience approaches has created an unprecedented opportunity for studying and understanding human psychiatric and neurological disorders. Each of these approaches offer a complementary set of tools to determine the causal relationship amongst multiple cellular and molecular pathways that govern intricate behavioral repertoires. Strategic initiatives, such as the Brain Research Through Advancing Innovative Neurotechnologies (BRAIN) Initiative (Ngai, 2022) and the Somatic Cell Genome Editing (SCGE) program (Saha et al., 2021), are creating even more opportunities to merge genetic, genomic, and behavioral approaches for basic science discovery and therapeutic opportunities. The combination of these techniques can now be successfully used to conduct large-scale *in vitro* and *in vivo* screens for multiple candidate genes (Kuhn et al., 2021). Together these advances not only make characterization of model organisms more thorough, but will help advance the field of molecular neuroscience toward discovery of the causes of and treatments for disorders that affect millions of individuals around the world.

## Author Contributions

All authors listed have made a substantial, direct, and intellectual contribution to the work, and approved it for publication.

## Conflict of Interest

The authors declare that the research was conducted in the absence of any commercial or financial relationships that could be construed as a potential conflict of interest.

## Publisher’s Note

All claims expressed in this article are solely those of the authors and do not necessarily represent those of their affiliated organizations, or those of the publisher, the editors and the reviewers. Any product that may be evaluated in this article, or claim that may be made by its manufacturer, is not guaranteed or endorsed by the publisher.
